# Hollow Pollen Shells to Enhance Drug Delivery

**DOI:** 10.3390/pharmaceutics6010080

**Published:** 2014-03-14

**Authors:** Alberto Diego-Taboada, Stephen T. Beckett, Stephen L. Atkin, Grahame Mackenzie

**Affiliations:** 1 Sporomex Ltd., Newland Avenue, Driffield YO25 6TX, UK; E-Mails: becketts2@btinternet.com (S.T.B.); sla2002@qatar-med.cornell.edu (S.L.A.); G.Mackenzie@hull.ac.uk (G.M.); 2 Hull York Medical School, University of Hull, Hull HU6 7RX, UK; 3 Department of Chemistry, University of Hull, Hull HU6 7RX, UK

**Keywords:** sporopollenin, exines, pollen, antioxidant, UV protection, porous shells, high loading, controlled release, bioavailability

## Abstract

Pollen grain and spore shells are natural microcapsules designed to protect the genetic material of the plant from external damage. The shell is made up of two layers, the inner layer (intine), made largely of cellulose, and the outer layer (exine), composed mainly of sporopollenin. The relative proportion of each varies according to the plant species. The structure of sporopollenin has not been fully characterised but different studies suggest the presence of conjugated phenols, which provide antioxidant properties to the microcapsule and UV (ultraviolet) protection to the material inside it. These microcapsule shells have many advantageous properties, such as homogeneity in size, resilience to both alkalis and acids, and the ability to withstand temperatures up to 250 °C. These hollow microcapsules have the ability to encapsulate and release actives in a controlled manner. Their mucoadhesion to intestinal tissues may contribute to the extended contact of the sporopollenin with the intestinal mucosa leading to an increased efficiency of delivery of nutraceuticals and drugs. The hollow microcapsules can be filled with a solution of the active or active in a liquid form by simply mixing both together, and in some cases operating a vacuum. The active payload can be released in the human body depending on pressure on the microcapsule, solubility and/or pH factors. Active release can be controlled by adding a coating on the shell, or co-encapsulation with the active inside the shell.

## 1. Introduction

**Figure 1:**
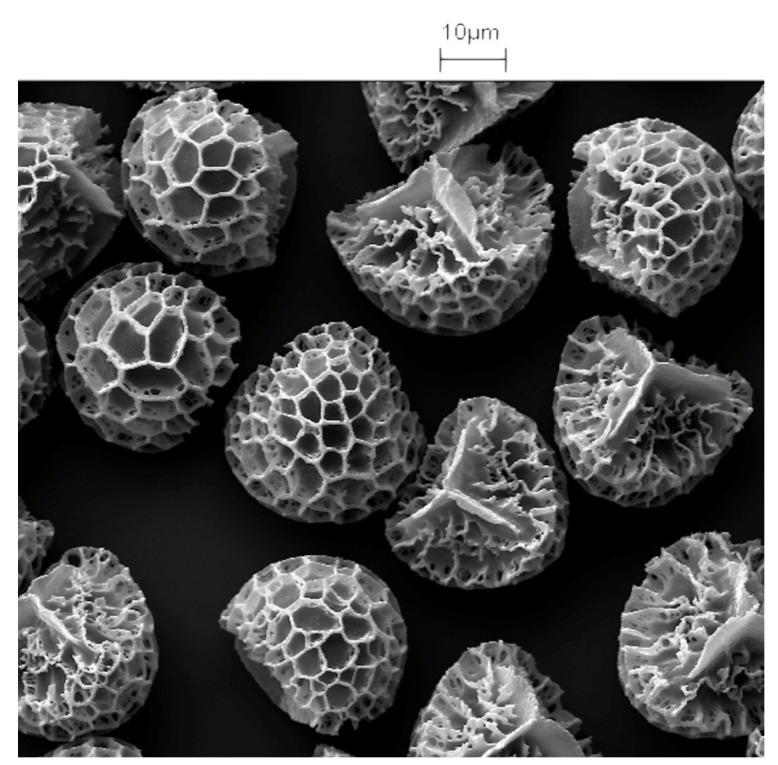
Scanning electron microscopy (SEM) picture of *Lycopodium clavatum* spores showing homogeneity of size and morphology.

Pollen particles, collected by bees and spores, for example, as obtained from *Lycopodium clavatum* (club moss), are regarded as being beneficial to health and can be purchased in most health food shops. All the particles from a single species of plant have the same size and decorations, for example *Lycopodium clavatum* particles shown in Figure 1. This morphology was regarded as being so applicable to drug delivery that artificial pollen particles have been produced using silica [1,2], calcium carbonate or calcium phosphate [3], and iron oxide [4], but these particles lack the elasticity of exines extracted from plant pollen and spores and are difficult to make free from defects. It is possible to attach drugs to the outside of complete particles, but the loading is restricted and the drug receives little protection and its release is relatively uncontrolled.

**Figure 2:**
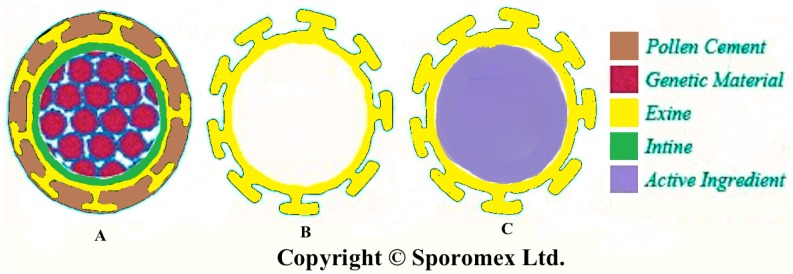
Schematic diagram illustrating Sporomex technology. (**A**) original spore; (**B**) empty shell; and (**C**) refilled shell.

For over ten years, the authors have developed a novel drug delivery system using the emptied shell of these particles and then refilling them with an active ingredient (Figure 2). In this way, the hollow centre gives a relatively high loading and the shell itself provides protection. The use of coating material inside or outside of the shell can give controlled release. The microencapsulated active can be used orally, by inhalation (very small particles only), or topically, and for other purposes, such as synthesis of peptides [5,6]; ion-exchange resin [7]; enhancing MRI (magnetic resonance imaging) images or micro reactors [8]. This paper however specifically deals with oral delivery.

Pollen grains are carriers of the plant’s genetic material needed for pollination [9] and plant spores are a part of the asexual plant reproduction. The external shells of such materials have many properties, such as good elasticity, physical and chemical resistance, UV (ultraviolet) shielding capability, and antioxidant activity. These properties enable the plant to protect its genetic material from external factors, such as oxidation by air, sunlight, and physical stress [10]. This shell has two distinct layers, the inner layer, called the intine, is composed largely of cellulose, and an external layer, called exine, which is mainly a polymer known as sporopollenin. This polymer is composed of carbon, hydrogen and oxygen [11] and, although its exact structure remains unknown, it has been described as an oxidative polymer of carotenoids [12] or polyunsaturated fatty acids [13,14,15,16]. There is also strong evidence for the presence of conjugated phenols [17]. The stability of sporopollenin is quite remarkable and pollen shells have been found by paleontologists in sedimentary rock a hundred of million years in age.

The genetic content of the spore or pollen and the inner layer of cellulose, if required can be removed through the porous shell wall [18,19] to give an empty shell. Any protein in the raw material is eradicated during the extraction to produce allergen free, non-toxic shells [20].

Pollens and spores are on the market as natural remedies [21] and nutraceuticals [22,23], whilst certain varieties have other uses, such as pyrotechnics. Ones, such as *Lycopodium clavatum*, *Helianthus annuus*, *Secale cereale*, *Aspergillus niger*, and *Chlorella vulgaris*, can be obtained in multi-ton quantities at a price that is commercially viable for many purposes. The extraction and filling processes use standard food processing machinery and are very simple compared with the manufacture of artificial pollen shaped particles.

Shells from the same plant species are very uniform in size, with different plants ranging in size as follows: *Myosotis* (Forget-me-not) (2.4–5 µm), *Chlorella vulgaris* (8–10 µm), *Brassica napus* (Oilseed rape) (10–12 µm), *Urtica dioica* (Nettle) (10–12 µm), *Ambrosia trifida* (Ragweed) (15 µm), *Phleum pratense* (Timothy grass) (22 µm), *Triticum* spp. (Wheat) (23 µm), *Cannabis sativa* (hemp) (25 µm), *Lycopodium clavatum* (25 µm), *Helianthus annuus* (Sunflower) (25 µm), *Lolium* L. (Rye grass) (40 µm), *Secale cereale* (Rye) (44 µm), *Pinus sylvestris* L. (Pine) (50 µm), *Zea mays* (Maize) (80 µm), *Cucurbita pepo* (Pumpkin) (200 µm) and *Cucurbita* (250 µm). The morphology of the shell is intrinsic to the source of the pollen or spore, the external decoration of the outer layer is a feature of the spore or pollen, as well as the empty shell from which it is extracted.

## 2. Extraction of Pollen Shells

The extraction of the pollen or spores can be carried out chemically, with enzymes, or a combination of both. The sporopollenin content depends upon the source of the pollen or spore with higher levels being preferred for producing intact shells.

### 2.1. Chemical Extraction

Early methods were designed to extract the sporopollenin by removing the other materials like lipids, cytoplasm, and the intine (cellulosic layer), in order to elucidate its structure. The first method, proposed by Zetzsche *et al.* [24,25,26,27], was based upon a sequential treatment with organic solvents to remove the lipids in the outer layer, which are responsible for the hydrophobicity of raw pollen and spores. Then, alkali was used to remove the genetic material from the inner cavity, followed by a final aqueous acid treatment to remove the polysaccharide intine. Subsequent studies [13,28,29,30,31,32,33,34] involved modifying the original method by introducing different acids or bases. Sporopollenin microcapsules have been obtained with similar extraction conditions [35] but using non-toxic mineral acids and alkalis make them more suitable for pharmaceutical applications.

One-pot methods have been described that involve the treatment pollen with strong acids. Tawashi has reported the use of 6 M hydrochloric acid at 110 °C for 24 h [36,37,38]. This process can be used for the extraction of shells from different types of pollens and spores, such as *Zea mays* (maize), *Secale cereale* (cereal rye), *Lycopodium clavatum* (club moss), *Pinus* spp. (pine), *Ambrosia trifida*, and *Ambrosia artemisifolia*. Another one-step method has been widely used to extract sporopollenin from a variety of pollens and spores [39] and reported by Erdtman [40]. This process involves removing lipids, polysaccharides, and proteins by acetolysis using a mixture of acetic anhydride and sulphuric acid.

Other chemical methods have been reported and include the use of anhydrous hydrofluoric acid in pyridine at 40 °C [41]; aqueous 4-methylmorpholine-*N*-oxide and sucrose under alkaline conditions at 20 °C or 70 °C for 1 h [42].

One-step treatment using sodium or potassium instead of strong mineral acids can produce shells with the cellulosic intine intact [35]. Some workers have used this double-layered microcapsule as an intermediate to obtain a single layered shell.

### 2.2. Enzymatic Extraction

A different approach to obtain sporopollenin is the use of enzymatic digestion of the contents of pollens and spores. In the literature several publications describe enzymatic methods to isolate the polymer. Different sequences of the enzymes (protease, lipase, amylase, pectinase, cellulases, and hemicellulose) can be used to remove the genetic content of the pollen grain together with the inner layer of cellulose, thereby avoiding the use of a strong acid. Following the sequence of enzymes used in gastric digestion together with a subsequent wash with hot methanol hollow exines have been produced from *Corylus avellana* L. (hazelnut) and *Pinus mugo* Turra (mountain pine) pollens [43,44,45,46,47].

## 3. Chemical Structure of Pollen Shells (Sporopollenin)

Sporopollenin has been defined as the main component of the outer layer exine of pollens and spores and the solid residue left after acetolysis [9]. The structure of sporopollenin remains imprecisely known, however, its elemental composition and functional groups have been accurately determined and found to be nitrogen-free [26]. The chemical structure of sporopollenin had been defined as an oxidative polymer of carotenoids [12] or polyunsaturated fatty acids [13,14,15,16]. Further studies, using a ^14^C-labelled oleic acid, showed it was incorporated into its structure [48]. Other unsaturated fatty acids, linoleic and linolenic, were found to be involved in its biosynthesis as precursors [14]. It was reported that since sporopollenin from different varieties of pollen and spores have similar IR (infrared) spectra, it is probable that the biosynthesis of the polymer has common pathways in each species [32,33]. More recently, it is been described as a mixture of co-polymers with an aliphatic core consisting of saturated and unsaturated oxygenated hydrocarbon [49,50]. Later studies of solid-state NMR (nuclear magnetic resonance) have reported the presence of unsaturated units involving a cross-linked aliphatic core in addition to aromatic side-chains [28,51]. FTIR (Fourier transform infrared) studies by Kawase *et al.* agreed with a high proportion of aliphatic units and suggested that possibly the aliphatic core of sporopollenin shares a common biosynthesis among all different taxa and vascular plants in particular [52,53]. This means that each type of plant produces different aromatic side chains, which could differentiate pollen grain and spores at a microscopic level by both morphology and chemical structure [43,54,55,56,57,58]. Further solid-state NMR studies provided more data with more detail on the structure showing that the core comprises of tetra-, hexa-, and octa-cosanes. Several studies have proposed the presence of short alkyl chains (16–18) in the skeleton of sporopollenin [59,60]. A number of analyses on different pollen species suggest a highly cross-linked structure and probably the presence of sterically hindered ethers, which taken together might explain the stability of sporopollenin toward harsh chemical treatment [14,54,61]. 

The polymer has a very strong lipophilic character, which is probably due the high presence of olefinic and aromatic groups along with the aliphatic character of the core. The presence of different types of hydroxyl groups, such as carboxylic acids, phenols, and alcohols however, infers some amphiphilic character to the polymer. Taken together, this gives the sporopollenin some very valuable properties which enable the empty shells to be loaded with actives with different ranges of polarities [62,63] and for them to be used as particle surfactants in the preparation of oil-water emulsions [64].

Initially, it was suggested that sporopollenin contains aromatic and then oxygenated aromatic units, such as phenols or ethers [32,33,65]. Later studies in the late 1980s, found *p*-coumaric and its derivatives following degradation of this polymer [46,47,66]. Further work using labelled precursors [43,67] and NMR spectroscopy indicated the presence of derivatives of cinnamic acids [68]. Other studies showed the importance of phenylalanine in the biosynthesis of sporopollenin to give phenyl propane unit, which remains intact in the final polymer [47,52,69]. The presence of phenolic groups in sporopollenin was showed using FTIR spectroscopy [69]. There are indications that phenols (particularly *p*-coumaric and ferulic acids) are not just involved as side chains within the core [52,70] but also in the cross-linking of the polymer [43,46,47,57,58,66,67].

Studies on *Lycopodium clavatum* spores, using ^14^C labelled acetic anhydride showed the presence of hydroxyl functional group in sporopollenin by acetylation [13,61,71,72]. The amount of ^14^C found on the polymer gave 6.6 hydroxyl groups per 90 C atoms [72]. 

Other functional groups like ketones were analysed by NMR, IR spectroscopy [69] and cytochemical studies but quantification was very difficult [49,50].

Wiermann *et al.* showed the presence of carboxylic acid and hydroxyl groups by spectroscopic analysis of enzymatic extracted sporopollenin [54,61]. Further investigation to produce primary amides by derivatisation with ammonia, gave the estimated loading which was quantified by combustion elemental analysis, as being between 1 and 2 mmol/g [71].

## 4. Physical and Chemical Properties of Pollen Shells

Pollen and spore shells are microcapsules that help the plant, moss, algae or fungi to protect its genetic material until pollination takes place. The manufacture of artificial shells for microencapsulation is usually costly whilst the homogeneity of the final product is not always the desired. Also they do not have all the properties of the natural plant material. The scale-up of these microcapsules is very difficult. Pollen and spore particles are produced by the plants to aid their sexual and asexual reproduction, respectively. They are monodispersed, which aids dispersion and optimises their role in plant reproduction. For example, pollen is required to be dispersed by wind, water, or insect to arrive at the female reproductive site of the plant. To transfer the genetic content, these shells are porous and also have an elasticity, which gives protection against impact. This is partly facilitated by the multidirectional nano-diameter-sized channels through the shell wall, which enables transport of water, nutrients and signaling agents across the wall. These nano-channels are used to empty the shells by the methods described previously to obtain the desired microcapsules. In addition, these nano-channels themselves can be used to encapsulate actives and potentially these shells can be used for drug delivery, although they have a much lower loading than the hollow centre. Bohne *et al.* estimated the diameter of the largest channels of the wall shells to be around 200–300 nm and indicated that the size and form of the nano-sized channels can be a restriction to the passage of actives, especially macromolecules [73].

One of the aims of the microencapsulation of an active is to preserve and therefore enhance its shelf-life. Sporopollenin, the main component of the exines, has evolved in pollen grains or spores to protect the genetic material from light and air oxidation, water, and insect damage. Pollen grains and spores can store their cytoplasm for very long periods, which shows the effectiveness of this protection. Rozema *et al.* reported the ability of sporopollenin to absorb sunlight and stated that these pollen walls could block effectively more than 80% of UV radiation [74]. Further studies describe a flat spectrum over the wavelength range 190–900 nm for the sporopollenin shells and an absorption coefficient of approximately 0.02 µm^−1^ for the microcapsules extracted from *Lycopodium clavatum* and *Ambrosia trifida* [75]. The optical transmission found for a single exine microcapsule was approximately 50% of light at 450 nm which changes minimally with the type of particle or the wavelength. Microcapsules obtained from spores and pollens of different sizes and species as for example *L. clavatum* (25 µm diameter), *Secale cereale* (45 µm diameter) and *Zea mays* (80 µm diameter); have all been able to prolong the shelf-life of commercial cod liver oil (Seven Seas, Hull, UK). The larger hollow shells have a bigger cavity and therefore are able to encapsulate more active. Such as *L. clavatum* spores and *Secale cereale* pollen are accessible in bulk quantities and shells extracted from these species have improved the stability and hence prolong the shelf-life of omega oil over a period of two months with lower peroxide values compared to the control [17,76]. In a series of experiments, in which sporopollenin encapsulated fish oil was irradiated with low energy UV irradiation for 2 h the peroxide values (PVs) of the encapsulated oil extracted from microcapsules was remarkably lower than that of the fish oil similarly treated without sporopollenin protection. In addition, the protection against the UV light was evident up to ratios of 1:6 (exines:oil; *w*/*w*), with all of the oil not being fully encapsulated.

All of these results indicate that sporopollenin can protect the oil in two different ways, the antioxidant properties of the shell as well as protection against UV light. Recent studies [17] to explore the oxidative electrochemistry of sporopollenin from different species, such as *L. clavatum* and *A. trifida*, revealed the presence of a mixture of conjugated phenol functionalities within the sporopollenin, which may explain the antioxidant properties and protection of the genetic material against UV light. Coumaric and ferulic acids, for instance, are present in the structure of the polymer [70] and are also known to protect against oxidation [77]. The antioxidant properties but in particular the light screening, make these shells beneficial when used in microencapsulation, particularly to protect light sensitive drugs.

The colour of the shells extracted by the methods described previously can vary from light brown/yellow to dark brown or black depending of the species involved [78]. The colour of the shell can be modified by bleaching with different chemical treatment, such as sodium hypochlorite or sodium chlorate. The colour of the shells can be measured by L* value—values go from 0 (pure black) to 100 (pure white). Shells extracted from different sources have been bleached to show an L* up to 90. Bleached shells can be dyed using either artificial or food colorants. A white colour is often required for many pharmaceutical and food uses as being cosmetically more acceptable.

## 5. Encapsulation and Release of an Active

### 5.1. Filling the Shell

The empty exine shells readily encapsulate an active substance due to the porosity of the shell walls. The active should be in a liquid form, either as a melt or solution. The active can be encapsulated by forming a suspension in the appropriated liquid and any solvent is subsequently removed by evaporation [62]. A limitation to the loading of actives in solution is the solubility of the active in the solvent, since the solvent itself takes up some of the volume within the shell. Higher loadings can be achieved using a series of filling, with more active being accumulated at each stage.

An alternative to this method is the encapsulation of two or more reagents, which react inside the shell to give a sparingly soluble or insoluble product. In this procedure way, the shell is acting like a micro-reactor [8].

The filling process often simply involves mixing the shells with the active in a liquid form and then applying a vacuum or centrifugation if needed. For aqueous solutions, a vacuum is frequently required and the solvent is removed by evaporation or freeze-drying. The shells have good elasticity and they can be compressed into a tablet form. If this is then placed in a liquid, the shells can recover their shape by absorbing it [8,35,76,79]. Industrial scale loading can be achieved using vacuum-tumbling, which is simpler in procedure and equipment than is required for spray-drying. Polar and non-polar materials with different molecular masses can be encapsulated into shells of *L. clavatum*, in particular, as well as with those obtained from *Zea mays* (maize), *Secale cereale* (cereal rye), *L. clavatum* (club moss), pine, *A. trifida*, and *A. artemisifolia*.

**Figure 3:**
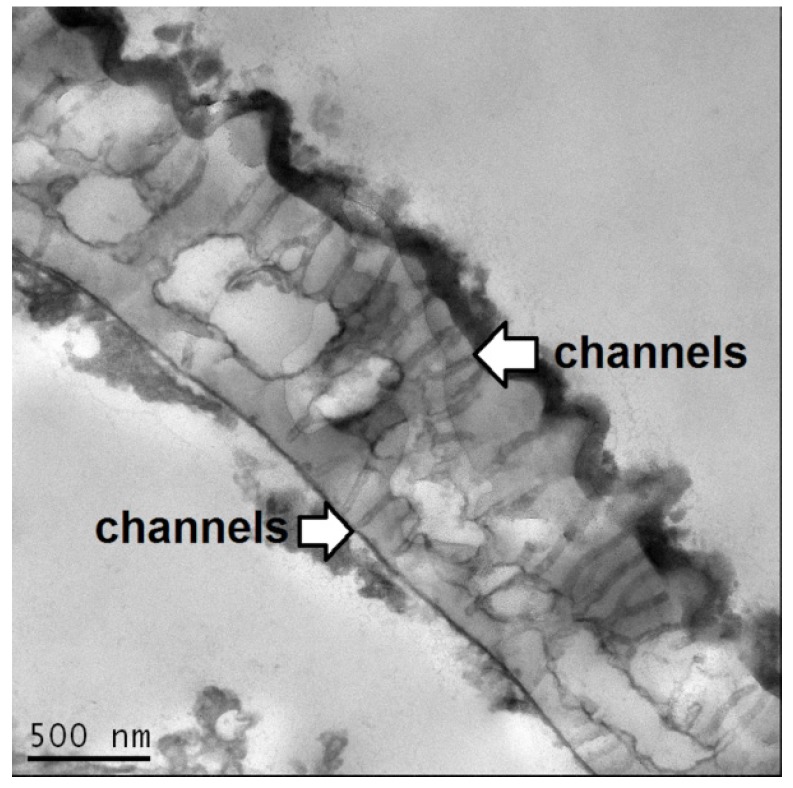
Transmission electron microscopy (TEM) picture of exine wall of *Secale cereale* showing nano-diameter (*ca.* 20 nm) sized channels.

Different techniques employed to fill the exines shells are now described in more detail:

The simplest method is passive encapsulation, which is where the shell and the active are mixed and the material is absorbed through the porous (Figure 3) walls into the inner cavity by capillary action [62]. A mixer is required, in order to obtain a homogenous sample. This can be a vortex mixer for small-scale samples or an industrial blender for kilogram size and above operations. Examples of this technique are the encapsulation of lipophilic liquids with low viscosity, e.g., cod liver oil and molten waxes, such as carnauba wax, beeswax, or molten mixture of fatty acids, such as cocoa butter [80]. Recent studies have reported that the shells are able to encapsulate material up to a loading of 75% and that with loadings below 50% the shells are able to mask the unpleasant flavour of these oils [81].

The passive loading can be aided by applying a vacuum of *ca.* 25 hPa. This is normally used for filling with more viscous materials such as syrups or waxes [62] or simply to speed up the processing. One example of this technique is the encapsulation of an ethanolic solution of ibuprofen. SEM (scanning electron microscopy) microscopic studies confirmed complete encapsulation of the drug. Double blind taste masking trials were performed with 50% ibuprofen-loaded exines and showed the ability of the shell to mask some of the pungent and bitter taste of the active [20].

Another study reported using vacuum to encapsulate dyes, such as malachite green, Evan’s blue, and Nile red with the encapsulated materials being assessed by CLSM (confocal laser scanning microscopy) [82]. This study also described the encapsulation of polar materials, such as enzymes (amylase, galactosidase, alkaline phosphatase and sHRP (horseradish peroxidase)) with loading around 20% (drug:drug and exine) but most significantly these experiments showed that the enzymes kept most of their catalytic activity when they were re-extracted from the shells [62].

Centrifugal aided filling, where the mixing of the active and the shells is followed by centrifugation (10,000× *g*) can be used for more viscous solutions/suspensions. Examples of this technique can be found in studies by Barrier *et al.*, including encapsulating a solution of alkaline phosphatase in glycerol at 4 °C with a 20% loading [62].

An alternative technique involves the compression of shells to give a cylindrical pellet. The dimensions of a typical pellet can be 0.5 cm deep by 1 cm diameter, which is equivalent to 1 g of shells compressed *ca.* 5 tonnes cm^−3^. The pellet can then be added to a solution of the active or the active as a melt [8,35]. Due to elasticity of the shells they recover virtually the same morphology as prior to compression and rapidly absorb the fluid.

### 5.2. Release of the Active Material

The encapsulated actives are released from the exines through the same nano-channels that penetrate the walls of the shells that had previously enabled the exines to be loaded. The controlled release of the active can be aided by several different techniques.

The release of the active can be achieved by passive diffusion. There are many factors in the surroundings of the loaded shells that can prompt this release, depending on the properties of the drug or protection employed. Where the active is a liquid the application of pressure on the elastic shell can result in a partial release, with 70%–80% emptying occurring after a series of pressings [63,83]. This is used for topical treatments.

The active can also be extracted from the loaded shells, by submerging them in a solvent in which the drug is soluble. X-ray diffraction (XRD) studies have shown that encapsulated vitamin D is crystalline inside the shell but can be released from the shell when it is agitated in ethanol. The ethanol goes through the nano-pores, dissolves the vitamin which can then diffuse out (publication in submission). Moreover, the release rate can be controlled by selecting the solvent according to the solubility of the active in it. Recent studies have also shown that changes in pH can trigger the release of ibuprofen in simulated gastrointestinal fluids [20]. For example, ibuprofen encapsulated in spore shells was fully released with a buffer of pH 7.4 but at pH below 1.5 almost 90% of the active is retained within the spore exine. In another study, it was reported that Omniscan™ (the magnetic resonance imaging (MRI) agent) was released much more rapidly in human blood than in buffer of pH 7.4. This was attributed to partial enzymatic digestion of the shells in blood [84]. 

**Figure 4:**
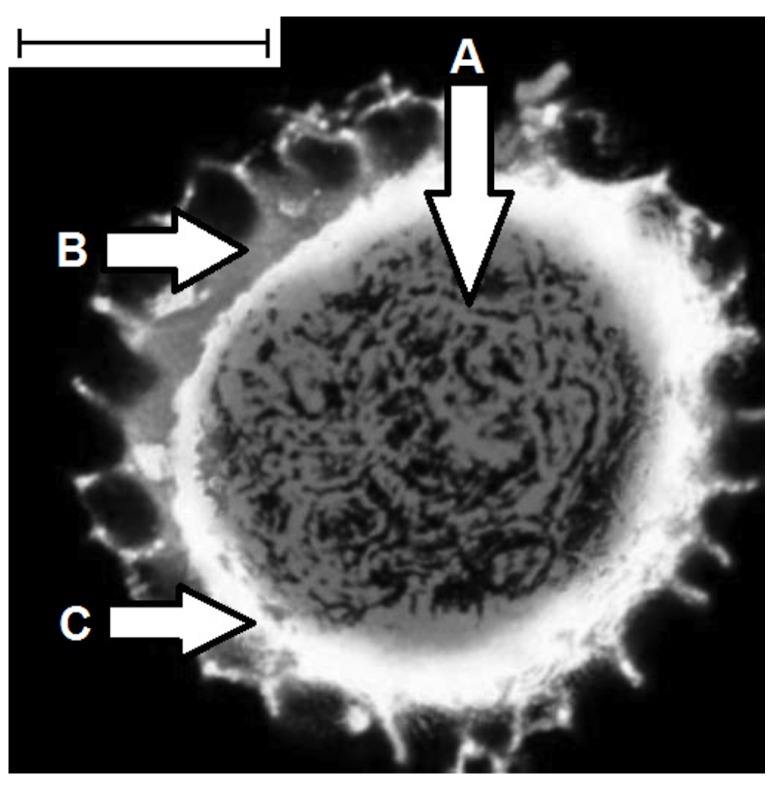
Confocal laser scanning microscopy image of a coencapsulated exine shell from a *L. clavatum* spore: (**A**) encapsulated Malachite green (the active); (**B**) exine wall; and (**C**) cocoa butter (coencapsulated). Scale bar: 60× oil immersion lens.

In addition to pH, the release of an encapsulated active can be triggered by adjustment of external conditions such as temperature and pressure. A further means to influence release is by making small modifications to the functional groups on the surface of the shells to alter its polarity and thereby the release profile of the active depending on its polarity. For example, carboxylic acids on the surface of the exines can be converted into their anionic salt forms, which will repel and help retain a non-polar active that is encapsulated within the exines. Secondly, it is possible to use a protective excipient, which can be added on to the surface of the exine shell as a coating or encapsulated with the active (“coencapsulant”) within the inner cavity. Two different means of achieving this coencapsulation are by encapsulating the active and the protective agent at the same time or by encapsulating the excipient after the active sequentially to create a protective layer between the active and the exine shell as shown in Figure 4. This also includes the filling of the pores of the shell wall itself. Potentially, coencapsulation has advantages over simple coating of the shell, which is more difficult to fully achieve and the external coating is more susceptible to damage or being removed. The use of excipients commonly applied in the pharmaceutical industry as protective additives have been successfully tried *in vitro* [80]. Examples of these additives are acrylate based polymers, such as Eudragit^®^, or cellulose based polymers, such as Aquacoat^®^, a cellulose acetate phthalate.

Eudragit^®^, a poly(meth)acrylate polymer soluble in organic solvents, has been used to protect a protein in simulated gastric fluid. After incubation in this fluid at body temperature, the microcapsule was able to retain up to 90% of the active. However, the protein was effectively released after 5 min following a pH change in phosphate buffer saline at pH 7.4. The range of excipients that can be used is wide and includes fats and oils (esters or acids), waxes, and saccharides and their polymers [80]. An example of these includes gum arabic, which is able to retain *in vitro* up to an 80% of an encapsulated protein over 45 min under simulated gastric fluid conditions. Other additives shown to be effective include cod liver oil/1% lecithin (60% protein retained), gelatine (80% protein retained), starch (55%), and cocoa butter (60% protein retained).

Fats, fatty acids, and waxes are additives whose release can be triggered by increasing the temperature. This implies that the protective excipient can be selected based on its melting point. Combining these ingredients can give an excipient with a final melting point below 37 °C so that the body temperature can trigger the release of the active. Obviously, a limitation of this technique is that the active will start to be released as soon as the loaded microcapsules are ingested.

## 6. Enhance Bioavailability

**Figure 5:**
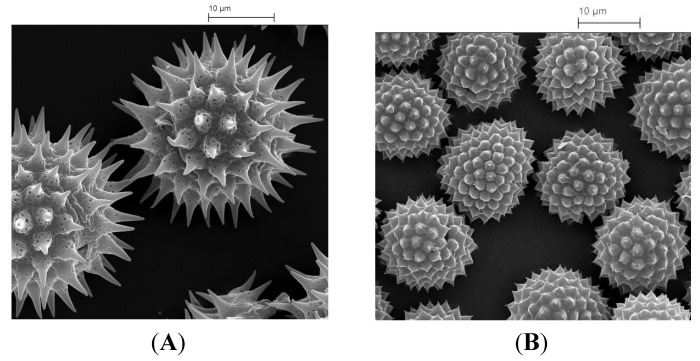
(**A**) Microcapsules extracted from sunflower pollen; and (**B**) microcapsules extracted from *Ambrosia trifida* pollen.

Improved bioavailability is important in the pharmaceutical industry in that it reduces costs and increases the effectiveness of drugs. Previous studies [35,85] have shown that this enhancement is possible using exines from pollens and plant spores. In one example the ingestion of ethyl ester derivative of EPA (eicosapentaenoic acid) encapsulated in sporopollenin microcapsules from *L. clavatum* spores improved the bioavailability of EPA in the bloodstream. In these trials, six volunteers ingested 4.6 g of fish oil containing 20% of the EPA ester, both alone, and encapsulated, as a 50% fat content powder, respectively. The bioavailability of the active was defined by area under curve (AUC (area under curve) 0–24 h). The mean AUC 0–24 of EPA from ethyl ester with exine was remarkably 10-fold higher than the corresponding for ethyl ester without exine encapsulation. In other unpublished studies involving human volunteers, ingesting a lipophilic active, the enhancement of bioavailability has been very similar. The mechanism by which such enhanced bioavailability is achieved is intriguing and some preliminary experiments have been undertaken to explore possible explanations. Recent *in vitro* studies (unpublished studies) revealed that the exine shells exhibited gut mucosal adhesion properties. In addition, *in vivo* experiments on mouse microtone sections of intestinal walls of mice fed with exines showed the exines to lie between the gut villi. The unique morphology of exine shell surfaces (Figure 5A,B) probably evolved to help pollen particles to become attached to insects and thereby aid the process of pollination. It is possible therefore that this same morphology could help the exine’s adherence to and close association with the gut wall leading to an increase in the period of contact between the encapsulated drug and the biological surface, thereby enhancing its absorption and bioavailability.

## 7. Summary

Microcapsules extracted from pollen and plant spores have several potential advantages over other methods of microencapsulation. They can be obtained from a natural and renewable source andthe processing is simple, inexpensive, and environmentally friendly.

Pollen and spore particles from the same species are equal in size, irrespective of where they have been cultivated and some sizes are available in multi-tonne quantities. After extraction and refilling the shells are still unchanged and homogeneous, which aids their dispersion when being further processed or ingested. Many pharmaceutical and food uses require a white powder that it will not be visible in the product. Although early extraction methods gave a dark coloured powder, further developments have produced whitened shells. 

The shells themselves are 1–2 µm thick and the emptied centre is able to encapsulate relatively large amounts of the active substance. The actual loading depends upon the active itself and the size of the shell, but a powder composed of fat in *L. clavatum* exines has been produced containing 80% of fat.

Sporopollenin, the main component of the shells, is very effective in protecting genetic material as well as the active substances with which it is refilled. This includes physical protection as well as heat—sporopollenin can withstand acids, alkalis and temperatures of 275 °C. In addition to providing protection of the active against oxidation initiated by UV and sunlight the shells have their own antioxidant properties that can be used to prolong shelf life of the active. Other microencapsulation systems, such as yeast, gelatin, or starch, do not have these antioxidant properties or UV protection and tend to have lower loadings especially yeast.

All forms of microencapsulation require the active to be released when it is at its most effective location. Work involving coating either the inside or the outside of the shell have shown that this can be achieved. 

A striking feature of the sporopollenin microcapsules is their ability to enhance bioavailability of actives *in vivo*, as shown in trials using human volunteers. Whilst the mechanism of such a phenomenon has yet to be verified, preliminary evidence is in favour of its being due to mucoadhesion, possibly influenced by the highly decorative features found on the exine shell walls. 

In conclusion, the use of shells extracted from pollen and plant spores to deliver drugs is at the development stage, but appears to offer considerable advantages over other microencapsulation techniques.
